# Optimization of PET reconstruction algorithm, SUV thresholding algorithm and PET acquisition time in clinical ^11^C-acetate PET/CT

**DOI:** 10.1371/journal.pone.0209169

**Published:** 2018-12-13

**Authors:** Sara Strandberg, Armin Hashemi, Jan Axelsson, Katrine Riklund

**Affiliations:** Department of Radiation Sciences, Diagnostic Radiology, Umea University, Umea, Sweden; Washington University School of Medicine, UNITED STATES

## Abstract

**Introduction:**

^11^C-acetate (ACE)-PET/CT is used for staging of high-risk prostate cancer. PET data is reconstructed with iterative algorithms, such as VUEPointHD ViP (VPHD) and VUEPoint HD Sharp IR (SharpIR), the latter with additional resolution recovery. It is expected that the resolution recovery algorithm should render more accurate maximum and mean standardized uptake values (SUV_max_ and SUV_mean_) and functional tumor volumes (FTV) than the ordinary OSEM. Performing quantitative analysis, choice of volume-of-interest delineation algorithm (SUV threshold) may influence FTV. Optimizing PET acquisition time is justified if image quality and quantitation do not deteriorate.

The aim of this study is to identify the optimal reconstruction algorithm, SUV threshold and acquisition time for ACE-PET/CT.

**Methods:**

ACE-PET/CT data acquired with a General Electric Discovery 690 PET/CT from 16 consecutive high-risk prostate cancer patients was reconstructed with VPHD and SharpIR. Forty pelvic lymph nodes (LNs) and 14 prostate glands were delineated with 42% and estimated threshold. SUV_max_, SUV_mean_, FTV and total lesion uptake were measured.

Default acquisition time was four minutes per bed position. In a subset of lesions, acquisition times of one, two and four minutes were evaluated.

Structural tumor volumes (STV) of the LNs were measured with CT for correlation with functional volumetric parameters.

To validate SUV quantification under different conditions with SharpIR 42%, recovery coefficients (RCs) of SUV_mean_ and FTV were calculated from a phantom with ^18^F-fluoro-deoxy-glucose (FDG)-filled volumes 0.1–9.2cm^3^ and signal-to-background (S/B) ratios 4.3–15.9.

**Results:**

With SharpIR, SUV_max_ and SUV_mean_ were higher and FTV lower compared with VPHD, regardless of threshold method, in both prostates and LNs.

Total lesion uptake determined with both threshold methods was lower with SharpIR compared with VPHD with both threshold methods, except in subgroup analysis of prostate targets where estimated threshold returned higher values.

Longer acquisition times returned higher FTV for both threshold methods, regardless of reconstruction algorithm. The FTV difference was most pronounced with one minute’s acquisition per bed position, which also produced visually the highest noise. SUV parameters were unaffected by varying acquisition times.

FTV with SharpIR 42% showed the best correspondence with STV.

SharpIR 42% gave higher RCs of SUV_mean_ and FTV with increasing phantom size and S/B-ratio, as expected.

**Conclusions:**

Delineation with SharpIR 42% seems to provide the most accurate combined information from SUV_max_, SUV_mean_, FTV and total lesion uptake. Acquisition time may be shortened to two minutes per bed position with preserved image quality.

## Introduction

Positron-emission tomography/computed tomography (PET/CT) is the current leading hybrid imaging method in oncology. The main indications are tumor staging, treatment planning, therapy response monitoring and detection of recurrent disease. PET/CT combines functional information from PET, depending on the selected radiotracer, and various structural anatomical information from the CT, depending on the selected imaging protocol. The CT is used in the range from low-dose organ localizer, to diagnostic-dose CT preferably with intravenously administered iodine contrast media to achieve optimal image quality.

^11^C-acetate (ACE) is suitable for prostate cancer (PC) imaging, being a marker for fatty acid synthase overexpression, which is present in PC cells [1]. The physiological ACE uptake is high in the pancreas, spleen and salivary glands, while the uptake is variable in the heart, muscles and skeleton. ACE is mainly excreted by the respiratory system and may only occasionally be seen in the urinary system, which is advantageous in the evaluation of structures in close relationship to the urinary bladder such as the prostate gland and pelvic lymph nodes (LN). Multiple studies have shown the value of ACE-PET/CT in staging of intermediate- and high-risk PC [2–4].

The maximum and mean standardized uptake values (SUV_max_ and SUV_mean_), functional tumor volume (FTV) and total lesion glycolysis (TLG) are four semi-quantitative PET parameters to aid in the evaluation of PET/CT, as prognostic markers and in therapy response monitoring with PET/CT over time [5]. The SUV scale represents the measured activity normalized to injected activity and patient body weight. SUV_max_ is the highest pixel in a defined volume of interest (VOI), and SUV_mean_ is the mean value of SUV of all pixels within a VOI. FTV is the volume of the VOI and TLG is the product of SUV_mean_ and FTV, combining metabolic and volumetric information. All these parameters were originally developed for ^18^F-fluoro-deoxy-glucose (FDG)—especially the term TLG obviously relates to FDG—but the principles and terminology can be applied also to other radiotracers. Since TLG is a frequently used term we chose to use TLG also for ACE total lesion uptake.

At Umea University Hospital, the currently used standard reconstruction algorithm for whole-body PET data in ACE-PET/CT and FDG-PET/CT acquired with GE Discovery 690 PET/CT scanner (General Electric, WI, US), is the Ordered Subsets Expectation Maximization (OSEM)-based iterative reconstruction (IR) algorithm VUEPoint HD ViP (VPHD) (General Electric, WI, US). VPHD may be replaced by another IR algorithm, VPHD Sharp IR (SharpIR) (General Electric, WI, US), which incorporates information about the PET detector response into the 3D IR algorithm and improves PET resolution [6].

SharpIR should render smaller FTV and TLG due to the improved resolution, and higher SUV_max_ and SUV_mean_ than VPHD, due to less impact from partial-volume effects.

Increased resolution might improve the diagnostic accuracy of ACE-PET/CT, but this remains to be proved in future clinical studies.

The thresholding algorithm also constitutes an important factor that influences image information. The VOI delineation is highly dependent on the chosen threshold level. A clinically most relevant methodological improvement of ACE-PET/CT would be to find the optimal thresholding method to achieve the most accurate FTV, to assist in radiotherapy treatment planning. Apart from the established 42% threshold method [7,8], an alternative estimated thresholding method is supplied for use with the GE Discovery 690 PET/CT scanner (General Electric, WI, US).

Another methodological feature is the shortening of PET data acquisition time, where shorter examinations would improve patient comfort, decrease the risk of patient movement artefacts, increase patient throughput per time unit and facilitate logistics for long-distance patients, while at the same time image information needs to be preserved. Due to the relatively fast kinetics of ACE, a whole-body scan does not reflect the same activity at different bed positions. A shorter image acquisition time may in fact allow a more comparable measure over a larger portion of the body.

The aim of this study is to identify the optimal PET reconstruction algorithm, SUV threshold algorithm and PET acquisition time in clinical ACE-PET/CT.

## Methods

### Patients

Sixteen consecutive high-risk PC patients examined with ACE-PET/CT for primary staging or biochemical recurrence between September 2014 and November 2014 were included. These patients were collected from a larger prospective study approved by the Regional Ethical Committee in Umea (approval number 2013-154-31). Verbal informed consent for participation and for publication was obtained from all study participants. The analyses were performed in accordance with the ethical standards of the Institutional Ethics Committee and with the 1964 Helsinki declaration and its later amendments.

The inclusion criteria of the current study were 1) biopsy-verified high-risk PC with >15% risk of pelvic LN spread and 2) pathological ACE uptake in the prostate gland and/or in pelvic LNs. The risk of LN spread was calculated from clinical pre-treatment nomograms [9], depending on parameters such as prostate-specific antigen (PSA) level, Gleason score and age. The average patient age was 68 years (median 66, range 56–82). 9/16 patients were referred for primary staging. 7/16 patients were referred for biochemical recurrence, after previous radiotherapy (4/7), radical prostatectomy (1/7) or androgen deprivation treatment (2/7). The patients in this study received radiotherapy and histopathological confirmation was not possible to obtain. Clinical patient data is presented in Table 1.

**Table 1:** Clinical patient data.

Patients (16)	Mean	Median	Range
Age (years)	68	66	56–82
PSA (ng/ml)*	75	44	5–330
Gleason score*	8	8	6–10
T stage**	T2	T2	T1c-T4
PSA relapse (ng/ml)	11	5	1–30

PSA—prostate-specific antigen.

*Data missing in 1/16 patients referred from regional hospital.

** Data missing in 2/16 patients referred from regional hospitals.

### Volume of interest parameters

SUV_max_, SUV_mean_, FTV and TLG were measured in clinical ACE-PET/CT in 54 VOIs, separated into two subgroups with 14 prostate glands and 40 pelvic LNs. PET data was reconstructed with VPHD and SharpIR and delineated with two thresholding methods: fixed 42% (relative SUV_max_) and estimated threshold. Structural tumor volumes (STV) of the LNs were analyzed by measuring the long diameter and two perpendicular axes in multiplane diagnostic CT images, and calculated from the formula for a spheroid. 37/40 LN targets could be evaluated in CT, and the remaining three were not distinguishable from surrounding tissues.

A subset of the ACE-PET/CT examinations (6/16) was recorded in list mode, which allowed for image reconstruction with acquisition times of one, two and four minutes per bed position. This was done in 18 lesions, with VOI analysis performed as above.

### Phantom

Classical phantom experiments (NEMA 2007 Image Quality Phantom) typically suffer from uptake defects from an approximately 1 mm thick plastic wall which separates the hot sphere from the lower-activity background. With high resolution imaging it becomes obvious that this gives an uptake profile that due to the non-radioactive wall deviates from that of a spherical lesion. Creating a phantom with close to non-existing wall thickness (less than 100 micrometer thickness) effectively gives an uptake profile that is very close to the uptake of a homogenous spherical lesion in a background. To simulate the various configurations of tumors and LN metastases, such a phantom was developed consisting of a 10 L plastic box, with seven different spherically shaped balloons reproducibly mounted from the lid. The balloons were filled with the same known radioactivity concentration, and their volumes were calculated from their mass. The balloon activity was held constant, while activity was added to the background between scans, to yield one scan with zero background, and six scans with signal-to-background (S/B) ratios: 2.1, 4.3, 6.5, 9.7, 13.4, and 15.9. After each addition of background activity, the water was thoroughly mixed using a propeller connected to a drilling machine. Due to radioactive decay, the activity concentration of the balloons decreased continuously throughout the experiment from 73.5 to 32.4 kBq/ml. The lowest S/B-ratio of 2.1 had to be excluded because the activity level was too close to background noise for the smaller balloons, leaving five S/B-ratios to be evaluated. In order to study smaller volumes than with the NEMA phantom, to simulate small LNs, neuro-intervention devices (Goldbal 3, Balt Extrusion, Montmorency, France) were used for the five smaller balloons numbered 1–5 (0.1, 0.2, 0.4, 0.5 and 0.7 cm^3^). The remaining two larger balloons, 6 and 7 (1.5 and 9.2 cm^3^), were made from two fingers of a rubber glove, tied with a knot. The five smallest balloons were spherical while the two larger were slightly elongated ellipsoids.

### PET/CT protocol and image analysis

All PET/CT examinations were acquired with a GE Discovery 690 PET/CT scanner (General Electric, WI, US). The patients were injected intravenously with 1-[^11^C]-acetate (4.0 MBq/kg body weight) and examined 10 minutes´ post-injection. The PET scan was performed in time-of-flight mode with an acquisition time of four minutes per bed position, from the proximal femur to the head.

PET images were reconstructed with two different algorithms. The first algorithm, VPHD, reconstructs images using the scanner implementation of the 3D iterative Ordered Subset Expectation Maximization (OSEM) reconstruction including time-of-flight, corrected for attenuation and scatter. The second reconstruction algorithm, SharpIR, is a more recent implementation that adds resolution recovery in the iterative reconstruction loop. VPHD was applied using two iterations and 24 subsets, and SharpIR was applied using three iterations and 24 subsets. The diagnostic contrast-enhanced CT covered the same part of the body with 50 cm field-of-view. The contrast media Omnipaque 350 mgI/ml was used at a dose of 0.5gI/kg body weight.

The parameters SUV_max_ (g/ml), SUV_mean_ (g/ml), FTV (cm^3^) and TLG (g·cm^3^/ml) were calculated using the PET-VCAR software in GE Advantage Workstation (GE, WI, US). This procedure includes manually defining a VOI search volume with a margin extending outside the PET uptake, followed by automatic delineation with either fixed 42%-threshold relative SUV_max_ (clinical default method), or with the estimated threshold algorithm supplied with PET-VCAR software. It was visually confirmed that only the intended structures were included.

The subset of data with varying acquisition times of one, two and four minutes per bed position gave three measurements of SUV_max_, SUV_mean_, FTV and TLG in 18 different VOIs at sufficient distance from the PET end slices, not to be affected by noise.

The phantom data was analyzed as above with regards to SUV_max_, SUV_mean_ and FTV. A true SUV was calculated from the known activities in the phantom. The SUV recovery coefficients (RC) were calculated as:

$${RC}_{SUVmax}=\frac{measured\;{SUV}_{max}}{true\;SUV}$$

$${RC}_{SUVmean}=\frac{measured\;{SUV}_{mean}}{true\;SUV}$$

RC_FTV_ was defined as:

$${RC}_{FTV}=\frac{measured\;FTV}{true\;V}$$

Phantom data was grouped within each S/B ratio in the different balloons, and average RCs of SUV_mean_ and FTV were calculated from all five S/B-ratios in all balloon sizes to imitate clinical scenarios with variable compositions, radiotracer uptake and size of metastatic LNs.

### Statistics

Observed differences in SUV_max_, SUV_mean_, FTV and TLG between SharpIR and VPHD reconstructions were evaluated with paired T-test. Paired T-tests were also used to evaluate differences between the two threshold algorithms, and between different acquisition times in clinical ACE-PET/CT.

Prostate lesions and suspected LN metastases were also evaluated separately in two subgroups. In both subgroup analyses, differences in SUV_max_, SUV_mean_, FTV and TLG were evaluated with paired T test, the same way as described above. In addition, observed differences in the subgroup data were evaluated and with related-samples Wilcoxon signed rank test because of the small subgroup sample size.

Correlation between FTV and STV was calculated with Pearson’s correlation coefficient.

The phantom data was evaluated with regards to average RC_SUVmean_, RC_SUVmax_ and RC_FTV_ in SharpIR with fixed 42% SUV_max_ threshold.

The statistical analyses were executed in IBM SPSS Statistics 22. Significance level was p<0.05.

## Results

### Differences between reconstruction algorithms SharpIR and VPHD in clinical ACE-PET/CT

SharpIR provided significantly higher SUV_max_ and SUV_mean_, and lower FTV and TLG, than VPHD. Details are listed below under each parameter.

#### SUV_max_

SharpIR produced 1.67 times higher average SUV_max_ (p<0.0001) than VPHD with both threshold methods. Separate subgroup analyses of prostate targets and LN targets showed similarly significant results in both tissues.

#### SUV_mean_

**Fig 1:**
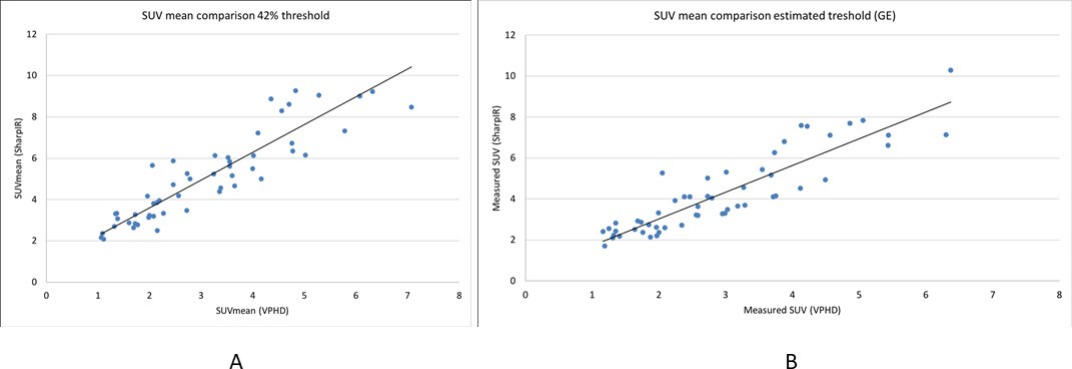
Diagrams of SUV_mean_ with 42% threshold and estimated threshold. (A) SUV_mean_ plotted for all lesions in VPHD and SharpIR clinical ACE-PET/CT, where ROIs are delineated with 42% threshold algorithm, and (B) estimated threshold algorithm. The black line is the best fit regression line.

SharpIR produced 1.35 times higher average SUV_mean_ than VPHD (p<0.0001) with 42% threshold, and 1.30 times higher with estimated threshold, as illustrated in Fig 1.

The SUV_mean_ range was 2.1–12.1 g/ml with SharpIR and 1.1–7.1 g/ml with VPHD with 42% threshold. The SUV_mean_ range with the estimated threshold was 1.7–10.3 g/ml with SharpIR and 1.2–6.4 g/ml with VPHD.

Also in separate subgroup analyses of prostate targets and LN targets, SUV_mean_ was significantly higher in SharpIR than in VPHD for both types of lesions. This difference appeared for both thresholding methods.

#### FTV

SharpIR 42% rendered the smallest average FTV (5.0 cm^3^), while the largest average FTV was acquired with VPDH with estimated threshold (16.2 cm^3^).

**Fig 2:**
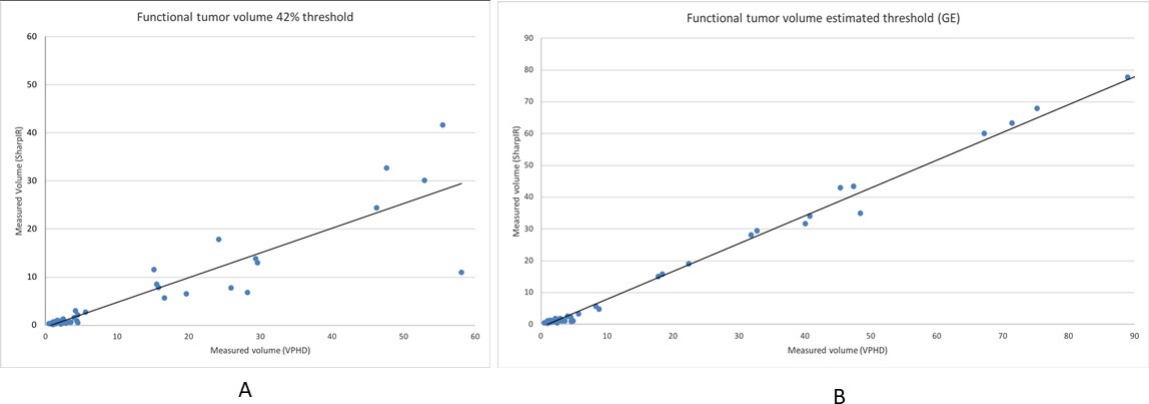
Diagrams of FTV with 42% threshold and estimated threshold. (A) FTV plotted for all lesions in VPHD and SharpIR clinical ACE-PET/CT, where ROIs are delineated with 42% threshold algorithm, and (B) estimated threshold algorithm. The black line is the best fit regression line.

Regardless of threshold algorithm, SharpIR produced significantly smaller average FTV than VPHD. All except for one of the FTVs were smaller with SharpIR than VPHD with 42% threshold (Fig 2).

FTV range was 0.5–58.1 cm^3^ with VPHD and 0.2–41.6 cm^3^ with SharpIR. Average FTV with VPHD was 1.58 times higher than with SharpIR with 42% threshold (p<0.0001), and 1.35 times higher with estimated threshold (p<0.0001). This was true also in separate subgroup analyses of prostates and LN targets, with both thresholding methods.

#### TLG

Analogous to the FTV results, average TLG was lowest in SharpIR with 42% threshold (25.5 g*cm^3^/ml), and highest in VPDH with estimated threshold (52.3 g*cm^3^/ml). TLG was significantly lower with SharpIR compared to VPHD with both threshold methods. This was true both in the analysis of all VOIs, and in subgroup analysis of the LNs. In subgroup analysis of the prostate targets, this was the case also with 42% threshold.

### Differences between 42% and estimated thresholding methods in clinical ACE-PET/CT

SUV_max_ was unaffected by thresholding methods. Differences between SharpIR and VPHD in SUV_mean_, FTV and TLG were more pronounced with 42% than with estimated threshold, in all but one VOI, where the FTV was higher with SharpIR than with VPHD.

### Differences between prostate and lymph node subgroups in clinical ACE-PET/CT

In separate subgroup analyses of prostates and LN lesions, all results were in accordance with the results from the data from the total number of lesions, except for TLG in prostate lesions, where there was no significant difference between SharpIR and VPHD with estimated threshold.

### Differences between different acquisition times in clinical ACE-PET/CT

No significant variations were seen in SUV_max_ and SUV_mean_ between the different acquisition times. FTV was significantly higher with longer acquisition times with both 42% threshold and estimated threshold. The difference in average FTV was more pronounced from 1 minute to 2 minutes, than from 2 minutes to 4 minutes’ acquisition per bed position. There was no significant difference between SharpIR and VPHD regarding FTV changes with different acquisition times. TLG was significantly higher in longer acquisition times with VPHD 42%, but this difference could not be seen with SharpIR. With estimated threshold, TLG was significantly higher in longer acquisition times with both VPHD and SharpIR.

**Fig 3:**
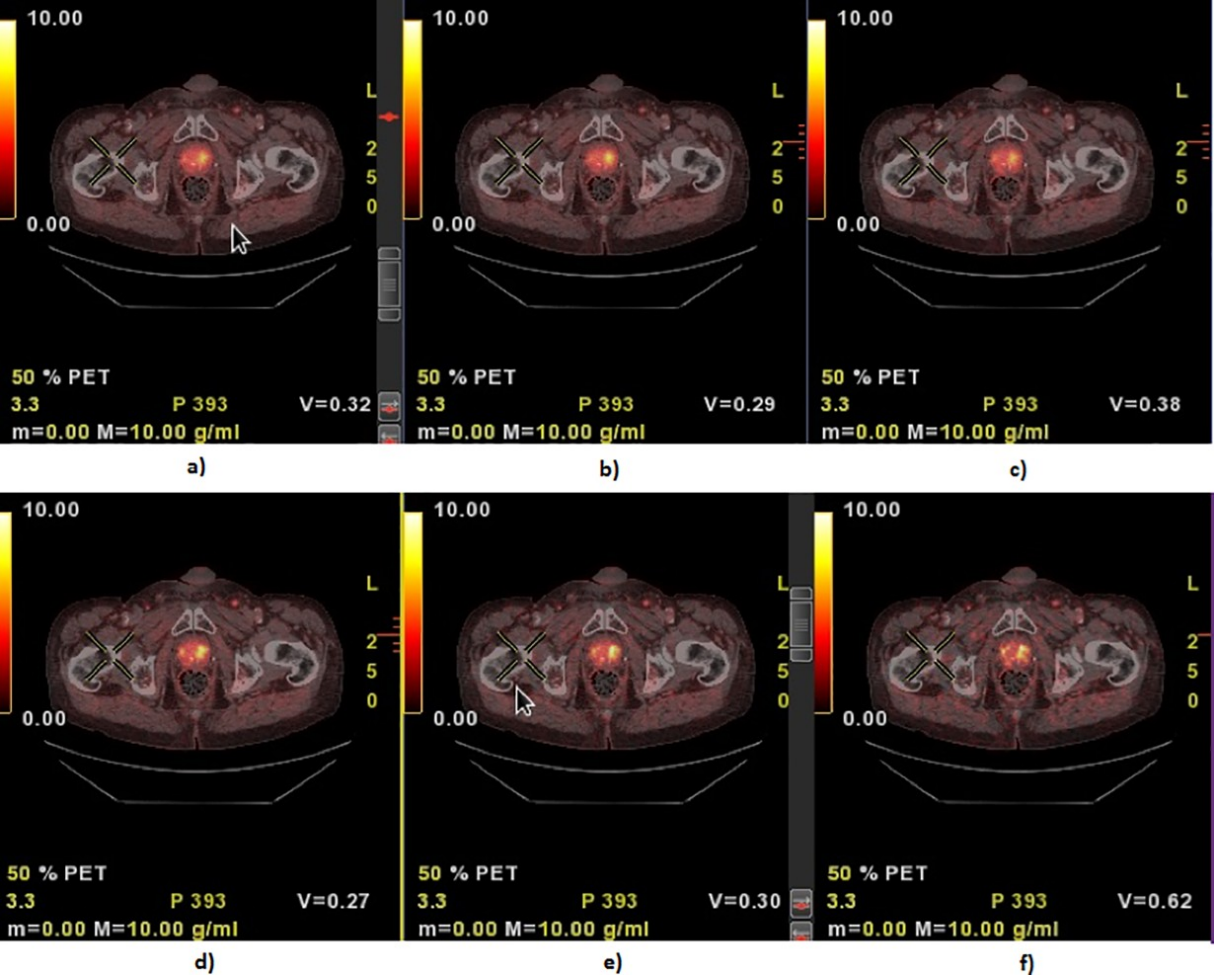
ACE-PET/CT with different acquisition times with VPHD and SharpIR. Transaxial PET/CT illustrating focal uptake in the left lobe of the prostate. (A, B, C) PET data reconstructed with VPHD 42% with 4, 2 and 1 minutes’ acquisition time, and (C, D, E) SharpIR 42% with 4, 2 and 1 minutes’ acquisition time.

Visually, signal-to-noise-ratio was impaired with acquisition time of 1 minute per bed position, compared to 2 and 4 minutes, which were both considered to yield acceptable diagnostic information, as illustrated in Fig 3.

### Comparison of FTV with STV in lymph nodes

**Fig 4:**
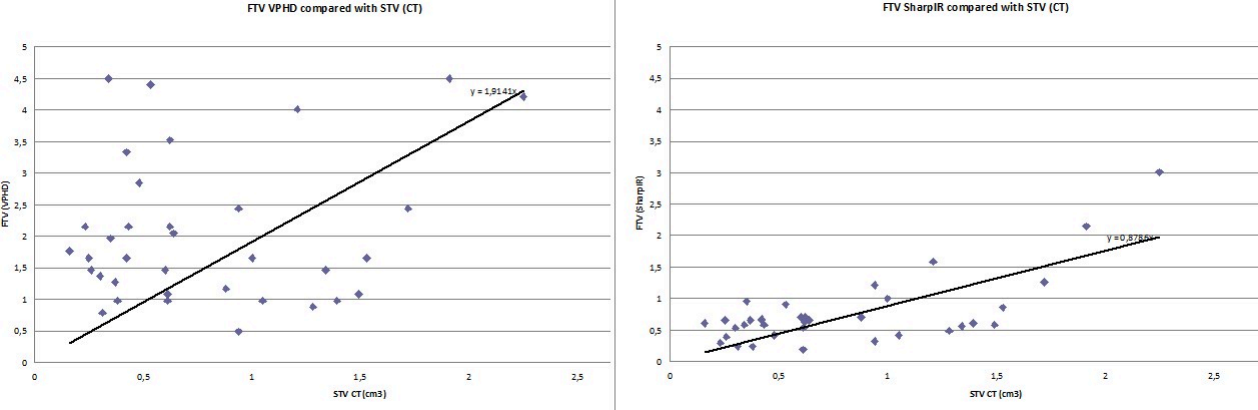
FTV compared to STV in LNs. FTV versus STV plotted for all LNs in VPHD (left) and SharpIR (right) in clinical ACE-PET/CT, with ROIs delineated with 42% threshold algorithm. The black lines represent the best linear regression, forced to include the origin.

For SharpIR 42%, FTV was a good approximation of STV. For VPHD 42%, FTV greatly overestimated the STV values (Fig 4), and a simple linear relation could not be identified.

Similar trends were observed with the estimated threshold (data not shown), but the 42% threshold yielded better correspondence between FTV and STV in LNs up to 2.5 cm^3^. In the two LNs > 2.5 cm^3^, FTV was smaller than STV.

On two occasions, two adjacent LNs in SharpIR were observed as one large LN with VPHD.

### Phantom RCs of SUV and FTV in SharpIR 42%

The average RCs of SUV_max_, SUV_mean_ and FTV were calculated from all balloons in each S/B-ratio, except for the smallest balloon 1 in S/B-ratio 4.3, which was unmeasurable and had to be excluded. Average RCs for all volumes in each S/B-ratio are presented in detail in Table 2. RCs were overall closer to 1 in higher S/B-ratios, as expected.

**Table 2:** Average RCs of SUV_mean_, SUV_max_ and FTV. For each S/B ratio, the average is calculated over all volumes.

S/B ratio	4.3	6.5	9.7	13.4	15.9
Average RC SUVmean	0.34	0.44	0.49	0.58	0.68
Average RC SUVmax	0.58	0.77	0.86	0.99	1.16
Average RC FTV	3.14	12.79	4.67	0.81	0.82

Average RCs for all S/B-ratios in each volume are presented in detail in Table 3. RCs were overall closer to 1 with increasing balloon size, as expected.

**Table 3:** Average RCs of SUV_mean_, SUV_max_ and FTV. For each volume, the average is calculated over all S/B ratios.

Volume (cm^3^)	0.1	0.2	0.4	0.5	0.7	1.5	9.2
Average RC SUVmean	0.22	0.33	0.48	0.57	0.61	0.65	0.65
Average RC SUVmax	0.31	0.57	0.78	0.91	0.98	1.03	0.95
Average RC FTV	27.63	3.58	1.32	1.36	0.72	0.54	0.87

**Fig 5:**
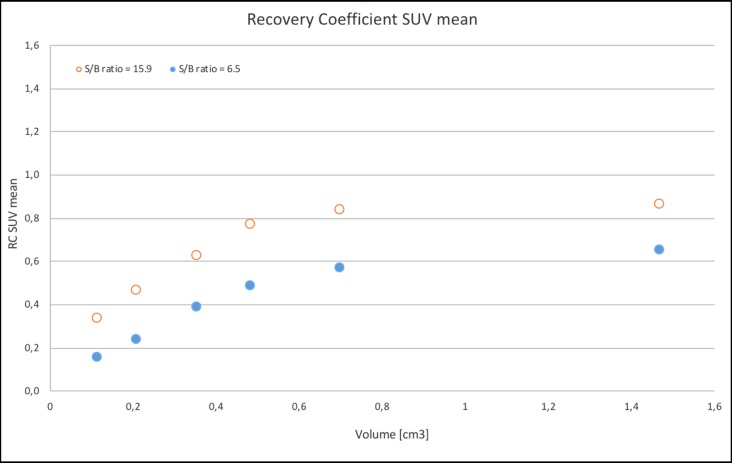
RCs of SUV_mean_. RC _SUVmean_ in different volumes. Red circles represent S/B-ratio 15.9 and blue dots S/B-ratio 6.5.

**Fig 6:**
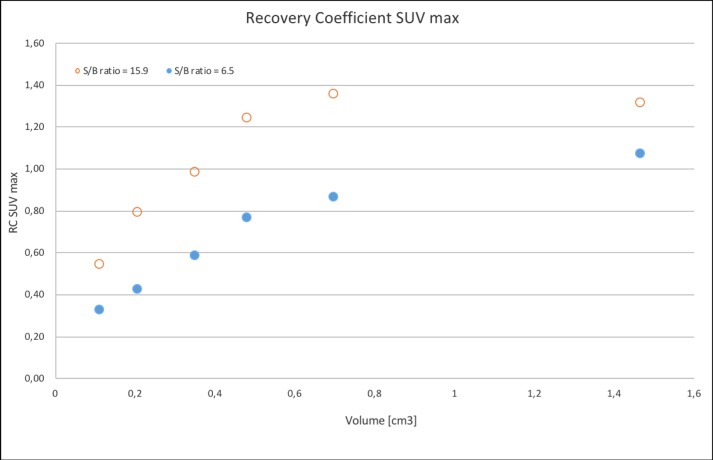
RCs of SUV_max_. RC _SUVmax_ in different volumes. Red circles represent S/B-ratio 15.9 and blue dots S/B-ratio 6.5.

Figs 5 and 6 illustrate a graphical example of RCs of SUV_mean_ and SUV_max_, respectively, in the different volumes, under two of the S/B-ratios.

## Discussion

SharpIR reconstruction algorithm rendered overall higher SUV_max_ and SUV_mean_, as well as lower FTV and TLG than VPHD in clinical ACE-PET/CT, as expected in our hypothesis.

The differences between SharpIR and VPHD were overall more pronounced with 42% than with estimated threshold, with very few exceptions. Those few outliers were almost exclusively found with the least robust parameter TLG and can be explained by the formula for TLG = SUV_mean_ * FTV. Small differences in SUV_mean,_ however not significant, have an impact on TLG, hence the slightly incongruent results.

It can be argued that the different number of iterations it the SharpIR and VPHD reconstructions may affect the results. It is known that for the same reconstruction method, increasing the number of iterations will increase SUV and decrease volume. However, the SharpIR has a much higher resolution than VPHD, which requires a larger number of iterations to achieve optimal balance between resolution and signal to noise. In this article, we have evaluated the effect of two provided clinical reconstruction methods with typically used clinical parameters. The imaging protocols are optimized to give correct quantification without sacrificing signal-to-background contrast or resolution, and we found that our preferred settings were the same as those recommended by the manufacturer.

In our study, SharpIR 42% showed the highest correlation with STV of LNs, a result which is in line with previous publications showing that fixed threshold values close to 41–42% give reasonable estimates of true volumes in solid tumors with volumes >4cm^3^ and S/B-ratios >5.42 [7, 8]. It is plausible to expect that the higher resolution in SharpIR compared to VPHD [7,8] is the reason why we see a good correlation in LNs well below 2.5 cm^3^. From Fig 4, the SharpIR data suggests that this correlation holds down to STV = 0.5 cm^3^, and at lower STV values the FTV values remain about 0.5 cm^3^.

The results from the subgroup analyses did not differ much from the results from data from all VOIs, see discussion above regarding outliers. A possible explanation for this could be that the subgroup samples were too small. Another reason could be that the ACE uptake itself does not differ between tissues.

The fact that we found a strong correlation between STV from CT measurements and the SharpIR 42% FTV suggests that the thresholding method is robust on group level. The reason why the volume correlation is better with SharpIR than VPHD is probably the improved resolution achieved with SharpIR. VPHD is limited by resolution issues especially when it comes to small structures like LNs, and as illustrated in Fig 6, variations in S/B-ratios render variations in SUV_max_ and FTV. In clinical scenarios, we therefore conclude that SharpIR should be the reconstruction method of choice.

However, it is not uncommon that individual tumors display large differences between STV and FTV. Generally, the FTV < STV for the larger LNs. The reason for this could be that only a fraction of the LN volume is affected by metastatic growth. For some LNs FTV > STV, which was more frequent in smaller volumes. It is unlikely that this reflects metastatic growth pattern and is probably due to flaws in the thresholding algorithm. We believe that for small LNs, the SUV_max_ becomes lower due to partial volume effects. Since the ROI is defined by the pixels above a SUV threshold value equal to 0.42 * SUV_max_, a lowered SUV_max_ would include more pixels. Thus, smaller LNs display apparently larger volumes with the 42% thresholding algorithm.

The influence of variations in acquisition time was evident in parameters FTV and TLG, which were higher with increased acquisition time and signal-to-noise-ratio. FTV and TLG in one minute´s images were the most variable, probably due to noise. There was also a qualitative difference, where especially one, but to some extent also two minutes’ images were visually noisier than the default four minutes’ images, which potentially might impair sensitivity, but this remains to be proved. Our familiarity with four minutes’ images might have had an influence on this subjective interpretation.

SUV parameters were not dependent on acquisition times in the observed interval of one to four minutes. Although these observations were limited to 18 ROIs, it seems reasonable to assume this holds true also in larger materials. Altogether, considering the trade-off between the desired amount of PET data information and the tolerable image acquisition time per examination, our conclusion is that it is possible to decrease image acquisition time to two minutes per bed position without affecting the quantification or reducing image quality.

An FDG-phantom was used as reference to verify the optimal method in clinical ACE-PET/CT, SharpIR 42%. We chose to calculate RCs for SUV_max_, SUV_mean_ and one volume parameter (FTV). TLG can be derived from SUV_mean_ and FTV. The phantom had been developed for another study [10] with the purpose of evaluating the combination of two algorithms for volume delineation and partial volume correction of uptakes in volumes smaller than 0.7 ml. It was noticed that RC for both FTV and SUV is highly dependent on both size and S/B ratio. The RCs of both SUV_mean_ and FTV tended to get higher with higher S/B-ratio and larger balloon size. The underestimation of RC for SUV is well known from many reports on the NEMA phantom. We do not find our results surprising for the even smaller volumes in the present phantom study.

The phantom measurements map the relation between lesion size and RC. In other words, if the size of a tumor is known, the measured SUV fraction of the actual tumor SUV can be estimated. An estimated RC can be used to obtain an RC-corrected SUV (SUVc) by SUVc = SUV / RC. This information could be used to set up a clinical tool with RC-corrected SUV depending on lesion size and S/B-ratio, however this was not within the aim of this project.

A possible limitation with this study could be that two different radiotracers were used, and that the quantitative parameters used were originally developed for FDG and were assumed to be compatible with ACE. However, the positron range of ^11^C and ^18^F are almost the same, and our decision to model ACE uptake in LN lesions using an FDG-filled phantom should not affect the results. Instead, we believe that the advantage of having a longer half-life and the fact that we therefore could use the same FDG-filled balloons throughout the phantom experiment, may have improved experimental accuracy.

Another limitation from a clinical point of view is the lack of histopathological verification of pathological uptakes.

SharpIR 42% improves PET information both in a quantitative and qualitative manner, but it remains to be proven whether it will affect sensitivity and specificity in staging of PC, and to evaluate the possible clinical impact. A direct clinical application of a more accurate estimation of FTV could be in radiotherapy planning to allow for dose painting, thereby having the potential to further personalize therapy and improve progression-free survival in high-risk PC patients.

## Conclusions

In conclusion, we recommend SharpIR 42% in clinical ACE-PET/CT, since that reconstruction method seems to render the most accurate combined information from SUV_max_, SUV_mean_, FTV and TLG. PET acquisition time can be shortened to two minutes per bed position with preserved image quality.

## Supporting information

S1 Appendix Anonymized data set PONE-D-17-38298. (XLS)

S2 Appendix Coronal maximum intensity projection PET image of the phantom. (TIF)
