# Supplementary figures and images for: Optimization of PET reconstruction algorithm, SUV thresholding algorithm and PET acquisition time in clinical 11C-acetate PET/CT
Source: PLoS One. 2018 Dec 13;13(12):e0209169. doi: 10.1371/journal.pone.0209169 (PMC6292629; doi:10.1371/journal.pone.0209169)

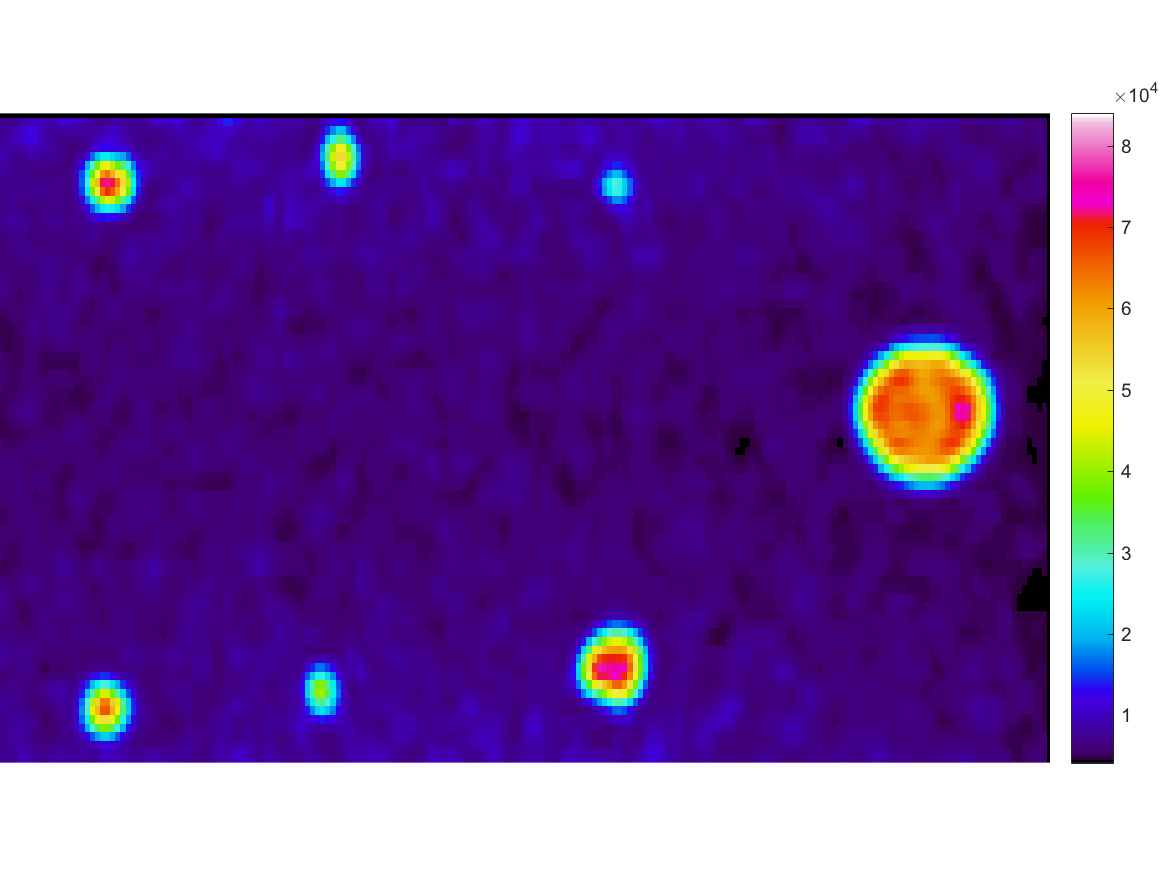

Supplement: S2 Appendix — (TIF) [file pone.0209169.s002.tif]
